# Associations between the *HLA-A *polymorphism and the clinical manifestations of Behcet's disease

**DOI:** 10.1186/ar3292

**Published:** 2011-03-24

**Authors:** Eun Ha Kang, Jeong Yeon Kim, Fujio Takeuchi, Joon Wan Kim, Kichul Shin, Eun Young Lee, Yun Jong Lee, Eun Bong Lee, Myoung Hee Park, Yeong Wook Song

**Affiliations:** 1 Division of Rheumatology, Department of Internal Medicine, Seoul National University Bundang Hospital, 166 Gumi-ro, Bundang-gu, Seongnam-si, Gyeonggi-do, Korea; 2 Division of Rheumatology, Department of Internal Medicine, Seoul National University Hospital, 28 Yeongeon-dong, Jongno-gu, Seoul, Korea; 3 Department of Internal Medicine, Faculty of Medicine, University of Tokyo, 7-3-1, Hongo, Bunkyo-ku, Tokyo, 113-0033, Japan; 4 Institute of Rheumatology, Medical Research Center, Seoul National University Hospital, 28 Yeongeon-dong, Jongno-gu, Seoul, Korea; 5 Department of Laboratory Medicine, Seoul National University Hospital, 28 Yeongeon-dong, Jongno-gu, Seoul, Korea

## Abstract

**Introduction:**

The objective was to investigate associations between the *HLA-A *gene and Behcet's disease (BD) and its clinical manifestations.

**Methods:**

Genotyping for the *HLA-A *locus was performed using the polymerase chain reaction-Luminex typing method in 223 BD patients and 1,398 healthy controls.

**Results:**

The phenotypic frequencies of HLA-A*02:07 (odds ratio (OR) = 2.03, *P *= 0.002), A*26:01 (OR = 1.85, *P *= 0.008), and A*30:04 (OR = 2.51, *P *= 0.006) tended to be higher in BD patients than in normal controls, but the frequency of A*33:03 (OR = 0.59, *P *= 0.003) tended to be lower in BD patients. A meta-analysis adopting our and the Japanese data confirmed the associations of HLA-A*02:07, A*26:01, and A*33:03 with BD. Furthermore, the frequencies of the HLA-A*02:07, A*26:01, and A*30:04 were significantly higher in patients with skin lesions (OR = 2.37, *P *< 0.0005, *Pc *< 0.012) and arthritis (OR = 2.32, *P *= 0.002, *Pc *= 0.048), with uveitis (OR = 3.01, *P *< 0.0005, *Pc *< 0.012), and with vascular lesions (OR = 9.80, *P *< 0.0005, *Pc <*0.012) and a positive pathergy test (OR = 4.10, *P *= 0.002, *Pc *= 0.048), respectively, than in controls. In HLA-B*51 non-carriers, these associations were also significant, being much stronger between HLA-A*26:01 and uveitis (OR = 4.19, *P *< 0.0005, *Pc *< 0.012) and between HLA-A*30:04 and vascular lesions (OR = 13.97, *P *< 0.00005, *Pc *< 0.0012). In addition, HLA-A*30:04 was associated with genital ulcers in HLA-B*51 non-carriers (OR = 3.89, *P *= 0.002, *Pc *= 0.048).

**Conclusions:**

HLA-A*02:07, A*26:01, and A*30:04 were associated with increased risk for BD, while HLA-A*33:03 with decreased risk. HLA-A*02:07, A*26:01, and A*30:04 were associated with skin lesions and arthritis, with uveitis, and with vascular lesions, genital ulcers, and a positive pathergy test, respectively.

## Introduction

Behcet's disease (BD) is a chronic relapsing inflammatory disease characterized by oro-genital ulcers, cutaneous inflammation, and uveitis. In addition to its typical muco-cutaneous and ocular manifestations, BD targets the musculoskeletal, vascular, nervous, and gastrointestinal systems [1]. Although the etiology of BD remains unclear, strong familial aggregations [2,3], a geographic distribution favoring the Middle East and East Asia [4], and the known association between BD and HLA-B*51 [4,5] indicate that genetic background importantly contributes to the pathogenesis of BD. In fact, HLA-B*51, the most prominent susceptibility gene [4,5], has been estimated to increase the relative risk of BD by 20% in the siblings of affected individuals [6], which suggests that other susceptibility loci exist. Candidate gene analyses have added a number of other genetic susceptibility loci for BD in and out of the MHC region [7-11]. However, the associations between the genes near MHC I region and BD are often doubted because of their linkage disequilibrium with HLA-B*51. On the other hand, recent genome-wide association studies (GWAS) have identified novel susceptibility loci across chromosomes [12-16] and *HLA-A *gene was shown to constitute a second independent susceptibility locus [14-16]. The *HLA-A *gene has been genotyped in BD patients with different ethnicities, and HLA-A*26 was reported to be associated with BD in Taiwan, Greece, and Japan [17-19]. In addition, a significant association between the HLA-A*26:01 subtype and BD was found in Japan [14]. In the present study, we genotyped the *HLA-A *gene in Korean BD patients and investigated the associations between its alleles and BD and the clinical features of BD.

## Materials and methods

### Patients and samples

Two hundred and twenty-three unrelated Korean patients who met the classification criteria proposed by the International Study Group for BD [20] were consecutively enrolled at Seoul National University Hospital. Medical records were reviewed for data regarding clinical manifestations. In addition to the data on oro-genital ulcers, skin and eye lesions, we collated data on arthritis based on joint swelling and pain, vascular involvement based on imaging studies (ultrasound, contrast-enhanced computed tomography, and/or angiography), central nervous system involvement based on cerebrospinal fluid examination, brain magnetic resonance imaging, and/or encephaloelectrography, and endoscopically identified gastrointestinal ulcerations. For controls, 1,398 subjects from unrelated hematopoietic stem cell donor registry of Korean Network for Organ Sharing (KONOS) were included. The individual demographic data of these controls were not made available to conceal personal information. Peripheral blood was collected from patients and controls after obtaining informed consent. This study was approved by the Institutional Board Review of Seoul National University Hospital (#0408-131-010) and patient consent was obtained.

### *HLA-A *and HLA-B*51 genotyping

Genomic DNA was extracted from peripheral blood using QIAamp blood kits (Qiagen, Valencia, CA, USA). The presence of HLA-B*51 was determined using polymerase chain reaction (PCR)-sequence specific primers; after amplifying a 581 base pair DNA fragment using the sequence specific primers 5'-GCCGGAGTATTGGGACCGGAAC-3' and 5'-CGGAGCCACTCCACGCACAG-3', nested PCR was performed using the sequence specific primers 5'-CTTACCGAGAGAACCTGCGGATCG-3' and 5'-CCGTCGTAGGCGTACTGGTT-3' [21]. *HLA-A *polymorphisms were examined by the PCR-Luminex typing method using a WAKFlow HLA typing kit (Wakunaga, Hiroshima, Japan) [22]. Briefly, after generic PCR amplification of the *HLA-A *region with biotinylated primers at the 5' end, the PCR amplicon was denatured and hybridized onto oligonucleotide probes immobilized on fluorescently-coded microsphere beads (Luminex, Austin, TX, USA) designed to specifically detect the nucleotide sequences of the PCR product at polymorphic sites of *HLA-A *gene. At the same time, the biotinylated PCR product was labeled with phycoerythrin-conjugated streptavidin and immediately examined using a Luminex 200 analyzer (Luminex). Genotype determination and data analysis were performed automatically using WAKFlow Typing software. Whenever atypical hybridization patterns were observed, samples were directly sequenced.

### Statistical analysis

Continuous values are presented as means ± standard deviations. The chi-square test or Fisher's exact test was used to compare the phenotypic frequencies of *HLA-A *alleles between patients and controls or between patients with and without certain clinical features. Statistical calculation was done using SPSS version 17.0 (SPSS Inc., Chicago, IL, USA). *P*-values of < 0.05 were considered significant. For multiple testings that compare patients and controls, Bonferroni correction was used to obtain corrected *P-*values (*Pc *value), and *Pc *values of < 0.05 were considered significant. Odds ratios (ORs) with 95% confidence intervals (CI) were estimated whenever applicable. For meta-analysis, data were pooled using Mantel-Haenszel method [23]. Between-study heterogeneity was quantified using the I^2 ^statistic [24]. The calculation was performed using RevMan software version 5.0 for Windows (Cochrane Collaboration, Oxford, UK).

## Results

### Clinical characteristics of BD patients

The clinical characteristics of the 223 BD patients are summarized in Table 1. Skin lesions (*n *= 180) included erythema nodosum (*n *= 130) and acneiform nodule (*n *= 105). Vascular involvement (*n *= 31) consisted of arterial pseudoaneurysm (*n *= 7), arterial stenosis (*n *= 1), valvulitis with or without aortitis (*n *= 3), and venous thrombosis (*n *= 26). Central nervous system involvement (*n *= 10) included brain parenchymal lesions (*n *= 6), aseptic meningitis (*n *= 2), seizure (*n *= 1), and cranial nerve palsy (*n *= 1). There was no case of gastrointestinal involvement. The HLA-B*51 allele was observed in 36.3% of patients and 20.2% of controls (OR = 2.26, *P *< 0.0000001).

**Table 1:** Demographic and clinical characteristics of 223 BD patients

Gender (M:F)	110:113
Age at diagnosis (years, mean ± SD)	43.1 ± 10.0
Disease duration (years, mean ± SD)	12.8 ± 9.2
Clinical manifestations	*n* (%)
Oral ulcer	223 (100)
Genital ulcer	159 (71.3)
Skin lesions	180 (80.7)
Positive pathergy test	94/182 (51.6)
Uveitis	85 (38.1)
Retinal vasculitis	10 (4.5)
Joint involvement	125 (56.1)
Vascular involvement	33 (14.8)
Central nervous system involvement	10 (4.5)
HLA-B*51†	81 (36.3)

†HLA-B*51 in controls = 282/1,398 (20.2%); *P *< 0.0000001; SD, standard deviation.

### Phenotypic frequencies of the *HLA-A *alleles

Thirty *HLA-A *alleles were observed either in patients or controls (Table 2). The phenotypic frequencies of HLA-A*02:07 (OR = 2.03, *P *= 0.002), A*26:01 (OR = 1.85, *P *= 0.008), and A*30:04 (OR = 2.51, *P *= 0.006) tended to be higher, whereas that of A*33:03 (OR = 0.59, *P *= 0.003) tended to be lower in patients than in controls.

**Table 2:** Distribution of phenotypic frequencies of *HLA-A *alleles

	All subjects				OR (95% CI)	*P* (*Pc*)	HLA-B*51 non-carriers				OR (95% CI)	*P* (*Pc*)	HLA-B*51 carriers				OR (95% CI)	*P* (*Pc*)
									
	BD *N *= 223		Control *N *= 1,398				BD *N *= 142		Control *N *= 1,116				BD *N *= 81		Control *N *= 282			
A*01:01	2	(0.9)	45	(3.2)	0.27 (0.07 to 1.13)		2	(1.4)	38	(3.4)	0.41 (0.10 to 1.70)		0	(0.0)	7	(2.5)	NA	
A*02:01	77	(34.5)	433	(31.0)	1.18 (0.87 to 1.58)		46	(32.4)	359	(32.2)	1.01 (0.70 to 1.47)		31	(38.3)	74	(26.2)	1.74 (1.04 to 2.93)	0.035 (> 1.0)
A*02:03	4	(1.8)	19	(1.4)	1.33 (0.45 to 3.93)		3	(2.1)	19	(1.7)	1.25 (0.36 to 4.26)		1	(1.2)	0	(0.0)	NA	
A*02:05	0	(0.0)	1	(0.1)	NA		0	(0.0)	1	(0.1)	NA		0	(0.0)	0	(0.0)	NA	
A*02:06	35	(15.7)	250	(17.9)	0.85 (0.58 to 1.26)		18	(12.7)	193	(17.3)	0.69 (0.41 to 1.17)		17	(21.0)	57	(20.2)	1.05 (0.57 to 1.93)	
A*02:07	27	(12.1)	89	(6.4)	2.03 (1.28 to 3.20)	0.002 (0.06)	19	(13.4)	80	(7.2)	2.00 (1.17 to 3.41)	0.010 (> 1.0)	8	(9.9)	9	(3.2)	3.32 (1.24 to 8.92)	0.031 (> 1.0)
A*02:10	0	(0.0)	13	(0.9)	NA		0	(0.0)	12	(1.1)	NA		0	(0.0)	1	(0.4)	NA	
A*03:01	3	(1.4)	35	(2.5)	0.53 (0.16 to 1.74)		3	(2.1)	28	(2.5)	0.84 (0.25 to 2.79)		0	(0.0)	7	(2.5)	NA	
A*03:02	1	(0.5)	6	(0.4)	1.05 (0.13 to 8.72)		1	(0.7)	5	(0.5)	1.58 (0.18 to 13.59)		0	(0.0)	1	(0.4)	NA	
A*11:01	39	(17.5)	242	(17.3)	1.01 (0.70 to 1.47)		25	(17.6)	188	(16.9)	1.05 (0.67 to 1.67)		14	(17.3)	54	(19.2)	0.88 (0.46 to 1.69)	
A*11:02	2	(0.9)	2	(0.1)	6.32 (0.89 to 45.08)		1	(0.7)	1	(0.1)	7.91 (0.49 to 127 to 13)		1	(1.2)	1	(0.4)	3.5 (0.22 to 56.58)	
A*24:02	95	(42.6)	578	(41.3)	1.05 (0.79 to 1.40)		54	(38.0)	442	(39.6)	0.94 (0.65 to 1.34)		41	(50.6)	136	(48.2)	1.10 (0.67 to 1.80)	
A*24:03	0	(0.0)	1	(0.1)	NA		0	(0.0)	1	(0.1)	NA		0	(0.0)	0	(0.0)	NA	
A*24:04	0	(0.0)	1	(0.1)	NA		0	(0.0)	1	(0.1)	NA		0	(0.0)	0	(0.0)	NA	
A*24:08	0	(0.0)	1	(0.1)	NA		0	(0.0)	1	(0.1)	NA		0	(0.0)	0	(0.0)	NA	
A*24:20	1	(0.5)	2	(0.1)	3.14 (0.28 to 34.82)		1	(0.7)	2	(0.2)	3.95 (0.36 to 43.84)		0	(0.0)	0	(0.0)	NA	
A*26:01	26	(11.7)	93	(6.7)	1.85 (1.17 to 2.93)	0.008 (0.24)	19	(13.4)	74	(6.6)	2.18 (1.27 to 3.72)	0.004 (0.12)	7	(8.6)	19	(6.7)	1.31 (0.53 to 3.23)	
A*26:02	11	(4.9)	58	(4.2)	1.20 (0.62 to 2.32)		7	(4.9)	46	(4.1)	1.21 (0.53 to 2.73)		4	(4.9)	12	(4.3)	1.17 (0.37 to 3.72)	
A*26:03	7	(3.1)	17	(1.2)	2.63 (1.08 to 6.42)	0.037 (> 1.0)	5	(3.5)	14	(1.3)	2.87 (1.02 to 8.10)	0.053	2	(2.5)	3	(1.1)	2.35 (0.39 to 14.34)	
A*26:05	0	(0.0)	1	(0.1)	NA		0	(0.0)	0	(0.0)	NA		0	(0.0)	1	(0.4)	NA	
A*26:18	0	(0.0)	1	(0.1)	NA		0	(0.0)	1	(0.1)	NA		0	(0.0)	0	(0.0)	NA	
A*29:01	2	(0.9)	28	(2.0)	0.44 (0.10 to 1.87)		2	(1.4)	24	(2.2)	0.65 (0.15 to 2.78)		0	(0.0)	4	(1.4)	NA	
A*29:02	1	(0.5)	0	(0.0)	NA		1	(0.7)	0	(0.0)	NA		0	(0.0)	0	(0.0)	NA	
A*30:01	8	(3.6)	74	(5.3)	0.67 (0.32 to 1.40)		7	(4.9)	63	(5.7)	0.87 (0.39 to 1.93)		1	(1.2)	11	(3.9)	0.31 (0.04 to 2.42)	
A*30:04	12	(5.4)	31	(2.2)	2.51 (1.27 to 4.96)	0.006 (0.18)	11	(7.8)	26	(2.3)	3.52 (1.70 to 7.29)	0.002 (0.06)	1	(1.2)	5	(1.8)	0.69 (0.08 to 6.01)	
A*31:01	22	(9.9)	160	(11.4)	0.85 (0.53 to 1.35)		10	(7.0)	98	(8.8)	0.79 (0.40 to 1.55)		12	(14.8)	62	(22.0)	0.62 (0.31 to 1.21)	
A*31:11	0	(0.0)	1	(0.1)	NA		0	(0.0)	0	(0.0)	NA		0	(0.0)	1	(0.4)	NA	
A*32:01	0	(0.0)	26	(1.9)	NA		0	(0.0)	25	(2.2)	NA		0	(0.0)	1	(0.4)	NA	
A*33:03	43	(19.3)	405	(29.0)	0.59 (0.41 to 0.83)	0.003 (0.09)	33	(23.2)†	345	(30.9)	0.68 (0.45 to 1.02)		10	(12.4)	60	(21.3)	0.52 (0.25 to 1.07)	
A*68:01	1	(0.5)	3	(0.2)	2.09 (0.22 to 20.23)		1	(0.7)	2	(0.2)	3.95 (0.36 to 43.85)		0	(0.0)	1	(0.4)	NA	

Values are presented as N (%). *P (Pc) *values are presented for those alleles with estimable OR (95% CI) and *P-*values of < 0.05.

†*P *= 0.047 (HLA-B*51 negative vs. positive patients). BD, Behcet's disease; CI, confidence intervals; NA, not applicable; OR, odds ratio; *Pc*, corrected *P*

When analyzed in HLA-B*51 non-carriers to exclude the effect of HLA-B*51 (Table 2), the frequencies of HLA-A*02:07 (OR = 2.00, *P *= 0.010), A*26:01 (OR = 2.18, *P *= 0.004) and A*30:04 (OR = 3.52, *P *= 0.002) tended to be more frequently observed in patients than in controls. There were no significant differences in the distribution of *HLA-A *alleles between HLA-B*51 negative and positive patients except for HLA-A*33:03, and its phenotypic frequency was lower in HLA-B*51 positive than in negative patients (*P *= 0.047).

We could not analyze gene-dose effects of these alleles on the risk of BD because all patients carrying HLA-A*02:07, HLA-A*26:01, HLA-A*30:04, or HLA-A*33:03 allele were heterozygotes except two patients with HLA-A*02:07 allele and one with HLA-A*33:03 allele.

### Meta-analysis of the case-control genetic association studies between *HLA-A *genes and BD susceptibility

To overcome the underpowered study design, a meta-analysis was performed. High resolution *HLA-A *genotyping data upon BD patients were only available for the Japanese population [14,25,26], thus Japanese data [14] were pooled together with ours using the allelic frequencies. Among 18 *HLA-A *alleles shared by Koreans and the Japanese, the frequencies of HLA-A*02:07, A*26:01, and A*26:03 were found to be higher and that of HLA-A*33:03 significantly to be lower in BD patients than in controls irrespective of HLA-B*51 status (Table 3). In addition, the frequency of HLA-A*26:02 was found to be higher in HLA-B*51 negative patients than in controls. The between-study heterogeneities were not significant for the above alleles. None of the Japanese individuals carried HLA-A*30:04 in the previously published studies [14,22,25,26].

**Table 3:** Meta-analysis on the association between *HLA-A *alleles and BD†

Allele	Total subjects					HLA-B*51 non-carriers				
	
	OR (95% CI)	*P*	I^2^ (%)	*P*_het_	Weight (%)	OR (95% CI)	*P*	I^2^ (%)	*P*_het_	Weight (%)
A*01:01	0.54 (0.21 to 1.42)	0.21	76	0.04	92.7	0.65 (0.22 to 1.91)	0.43	60	0.11	92.7
A*02:01	1.20 (0.97 to 1.48)	0.09	0	0.70	65.7	1.13 (0.87 to 1.47)	0.37	0	0.33	70.5
A*02:03	0.96 (0.35 to 2.65)	0.93	27	0.24	67.5	0.99 (0.32 to 3.09)	0.99	0	0.47	70.9
A*02:06	0.86 (0.66 to 1.13)	0.28	0	0.69	58.2	0.73 (0.50 to 1.06)	0.10	0	0.62	60.7
A*02:07	1.96 (1.34 to 2.85)	0.0005	0	0.62	66.6	2.00 (1.30 to 3.09)	0.002	0	0.69	69.7
A*02:10	0.37 (0.06 to 2.37)	0.30	0	0.43	80.0	0.37 (0.05 to 3.05)	0.36	0	0.72	74.5
A*03:01	0.44 (0.15 to 1.26)	0.13	0	0.56	71.1	0.77 (0.26 to 2.21)	0.62	0	0.84	75.7
A*03:02	0.71 (0.12 to 4.21)	0.70	0	0.56	52.4	1.12 (0.18 to 6.77)	0.90	0	0.64	51.9
A*11:01	0.78 (0.60 to 1.03)	0.08	77	0.04	50.5	0.85 (0.61 to 1.19)	0.35	51	0.15	50.6
A*11:02	1.30 (0.30 to 5.57)	0.73	75	0.05	18.0	1.23 (0.20 to 7.71)	0.82	57	0.13	11.5
A*24:02	0.89 (0.75 to 1.05)	0.18	44	0.18	47.0	0.83 (0.67 to 1.03)	0.10	0	0.43	48.8
A*24:20	0.60 (0.13 to 2.83)	0.52	59	0.12	12.1	0.68 (0.12 to 3.73)	0.65	63	0.10	12.6
A*26:01	1.89 (1.41 to 2.53)	<0.0001	0	0.91	38.9	2.42 (1.73 to 3.39)	<0.00001	0	0.62	41.3
A*26:02	1.48 (0.90 to 2.42)	0.12	7	0.30	64.5	1.93 (1.09 to 3.40)	0.02	69	0.07	69.1
A*26:03	2.01 (1.14 to 3.56)	0.02	0	0.51	28.4	2.40 (1.17 to 4.91)	0.02	0	0.70	36.6
A*26:05	3.69 (0.44 to 31.24)	0.23	0	0.69	45.3	NA				
A*31:01	1.22 (0.91 to 1.62)	0.18	79	0.03	51.9	0.73 (0.45 to 1.19)	0.21	0	0.76	51.8
A*33:03	0.52 (0.39 to 0.70)	<0.00001	64	0.09	71.0	0.58 (0.41 to 0.81)	0.001	67	0.08	71.3

†Genetic data were pooled using allelic frequency.

CI, confidence interval; I^2^, between-study heterogeneity; NA, not applicable; OR, odds ratios for the risk to develop BD; *P*, *P-*values for significance of each *HLA-A *allele in the pooled genetic effect (calculated by Mantel-Haenszel fixed method); *P*_het_, *P *values for heterogeneity statistics; weight (%), weight of the present study.

### Associations between *HLA-A *alleles and clinical features of BD

The phenotypic frequencies of HLA-A*02:07, A*26:01, A*30:04, or A*33:03 alleles were compared between a subset of patients having a particular clinical manifestation (genital ulcers, skin lesions, positive pathergy test, uveitis, arthritis, or vascular lesions) and controls (Table 4). It was found that the HLA-A*02:07 was associated with skin lesions (OR = 2.37, *P *< 0.0005, *Pc *< 0.012) and arthritis (OR = 2.32, *P *= 0.002, *Pc *= 0.048), A*26:01 with uveitis (OR = 3.01, *P *< 0.0005, *Pc *< 0.012), and A*30:04 with vascular lesions (OR = 9.80, *P *< 0.0005, *Pc *< 0.012) and positive pathergy test (OR = 4.10, *P *= 0.002, *Pc *= 0.048). *HLA*-A*33:03 was not associated with any particular manifestations. To further validate the associations between these *HLA-A *alleles and certain clinical manifestations, we compared the frequencies of HLA-A*02:07, A*26:01, and A*30:04 between patients with and without a specific clinical manifestation (Table 4). The frequency of A*26:01 was higher in patients with uveitis than without (OR = 2.47, *P *= 0.029) and that of A*30:04 in patients with vascular lesions than without (OR = 6.81, *P *= 0.003). The frequency of A*02:07 was only marginally higher in patients with skin lesions than without (OR = 3.31, *P *= 0.095).

**Table 4:** Associations between *HLA-A *alleles and clinical manifestations of BD

HLA alleles	Group	Phenotypic frequency *n *(%)	OR (95% CI)	*P*	*Pc*
All subjects					
A*02:07	Patients with skin lesions (*n *= 180)	25 (13.9)			
	vs. Patients without skin lesions (*n *= 43)	2 (4.7)	3.31 (0.75 to 14.54)	0.095	
	vs. Controls (*n *= 1,398)	89 (6.4)	2.37 (1.48 to 3.31)	<0.0005	<0.012
	Patients with arthritis (*n *= 125)	17 (13.6)			
	vs. Patients without arthritis (*n *= 98)	10 (10.2)	1.39 (0.60 to 3.18)	0.438	
	vs. Controls (*n *= 1,398)	89 (6.4)	2.32 (1.33 to 4.03)	0.002	0.048
A*26:01	Patients with uveitis (*n *= 85)	15 (17.7)			
	vs. Patients without uveitis (*n *= 138)	11 (8.0)	2.47 (1.08 to 5.68)	0.029	
	vs. Controls (*n *= 1,398)	93 (6.7)	3.01 (1.66 to 5.46)	<0.0005	<0.012
A*30:04	Patients with vascular lesions (*n *= 33)	6 (18.2)			
	vs. Patients without vascular lesions (*n *= 190)	6 (3.2)	6.81 (2.05 to 22.66)	0.003	
	vs. Controls (*n *= 1,398)	31 (2.2)	9.80 (3.78 to 25.43)	<0.0005	<0.012
	Patients with genital ulcers (*n *= 159)	10 (6.3)			
	vs. Patients without genital ulcers (*n *= 64)	2 (3.1)	2.08 (0.44 to 9.77)	0.516	
	vs. Controls (*n *= 1,398)	31 (2.2)	3.00 (1.42 to 6.16)	0.006	0.14
	Patients with positive pathergy test (*n *= 94)	8 (8.5)			
	vs. Patients with negative pathergy test (*n *= 88)	3 (3.4)	2.19 (0.74 to 6.46)	0.147	
	vs. Controls (*n *= 1,398)	31 (2.2)	4.10 (1.83 to 9.20)	0.002	0.048
HLA-B*51 non-carriers					
A*02:07	Patients with skin lesions (*n *= 109)	17 (15.6)			
	vs. Patients without skin lesions (*n *= 33)	2 (6.1)	2.86 (0.63 to 13.10)	0.243	
	vs. Controls (*n *= 1,116)	80 (7.2)	2.39 (1.36 to 4.21)	0.002	0.048
	Patients with arthritis (*n *= 83)	14 (16.9)			
	vs. Patients without arthritis (*n *= 59)	5 (8.5)	2.19 (0.74 to 6.46)	0.147	
	vs. Controls (*n *= 1,116)	80 (7.2)	2.63 (1.42 to 4.87)	0.002	0.048
A*26:01	Patients with uveitis (*n *= 48)	11 (22.9)			
	vs. Patients without uveitis (*n *= 94)	8 (8.5)	3.20 (1.19 to 8.59)	0.017	
	vs. Controls (*n *= 1,116)	74 (6.6)	4.19 (2.05 to 8.54)	<0.0005	<0.012
A*30:04	Patients with vascular lesions (*n *= 24)	6 (25.0)			
	vs. Patients without vascular lesions (*n *= 118)	5 (4.2)	7.53 (2.08 to 27.28)	0.003	
	vs. Controls (*n *= 1,116)	26 (2.3)	13.97 (5.13 to 38.08)	<0.00005	<0.0012
	Patients with genital ulcers (*n *= 106)	9 (8.5)			
	vs. Patients without genital ulcers (*n *= 36)	2 (5.6)	1.58 (0.32 to 7.67)	0.730	
	vs. Controls (*n *= 1,116)	26 (2.3)	3.89 (1.77 to 8.54)	0.002	0.048
	Patients with positive pathergy test (*n *= 57)	7 (12.3)			
	vs. Patients with negative pathergy test (*n *= 58)	3 (5.2)	2.57 (0.63 to 10.47)	0.203	
	vs. Controls (*n *= 1,116)	26 (2.3)	5.87 (2.43 to 14.17)	0.001	0.024

CI, confidence interval; OR, odds ratio; *Pc*, *P-*values corrected for multiple testing.

### Associations between *HLA-A *alleles and clinical features of BD in HLA-B*51 non-carriers

To eliminate the effect of HLA-B*51 on the clinical manifestations of BD (Additional file 1), the analysis was performed in HLA-B*51 non-carriers (Table 4). HLA-A*02:07 was associated with skin lesions (OR = 2.39, *P *= 0.002, *Pc *= 0.048) and arthritis (OR = 2.63, *P *= 0.002, *Pc *= 0.048), A*26:01 with uveitis (OR = 4.19, *P *< 0.0005, *Pc *< 0.012), and A*30:04 with vascular lesions (OR = 13.97, *P *< 0.00005, *Pc *< 0.0012), genital ulcers (OR = 3.89, *P *= 0.002, *Pc *= 0.048), and a positive pathergy test (OR = 5.87, *P *= 0.001, *Pc *= 0.024); the associations between HLA-A*26:01 and uveitis and between HLA-A*30:04 and vascular lesions were much stronger in HLA-B*51 negative patients than in total patients. *HLA*-A*33:03 was not associated with any particular manifestations.

The frequency of HLA-A*26:01 was higher in patients with uveitis than without (OR = 3.20, *P *= 0.017) and that of HLA-A*30:04 in patients with vascular lesions than without (OR = 7.53, *P *= 0.003).

### Distribution of clinical manifestations according to HLA-B*51 and *HLA-A *status

Because not only *HLA-A *alleles but also HLA-B*51 seemed to be associated with skin lesions or uveitis (Table 4, Additional file 1), we stratified the occurrence of skin lesions or uveitis according to the presence or absence of HLA-B*51 and particular *HLA-A *alleles to better assess the independent effect of HLA-A*02:07 and A*26:01 and their genetic interaction with HLA-B*51 on these clinical manifestations (Table 5). There was a trend that HLA-B*51 and HLA-A*02:07 are additive to increase the risk of skin lesions, which, however, was not statistically significant, probably due to the limited power of analysis. While both HLA-B*51 and HLA-A*26:01 seemed to be risk factors for uveitis, the risk to uveitis was not escalated with the combination of HLA-B*51 and HLA-A*26:01 than with either one of the two alleles.

**Table 5:** Distribution of clinical manifestations according to HLA-B*51 and *HLA-A *status

Patient subset	*n *(%) of clinical manifestations	OR (95% CI)	*P*
	Skin lesions		
B*51+ A*02:07+	8/8 (100)	NA	0.20
B*51- A*02:07+	17/19 (89.5)	2.86 (0.63 to 13.10)	0.24
B*51+ A*02:07-	63/73 (86.3)	2.12 (0.97 to 4.64)	0.055
B*51- A*02:07-	92/123 (74.8)	(referent)	
	Uveitis		
B*51+ A*26:01+	4/7 (57.1)	3.10 (0.66 to 14.53)	0.21
B*51- A*26:01+	11/19 (57.9)	3.20 (1.19 to 8.59)	0.017
B*51+ A*26:01-	33/74 ((44.6)	1.87 (1.03 to 3.40)	0.039
B*51- A*26:01-	37/123 (30.1)	(referent)	

CI, confidence intervals; NA, not applicable; OR, odds ratio.

## Discussion

The present study shows that three *HLA-A *alleles, A*02:07, A*26:01, and A*30:04 might be BD susceptibility alleles, while A*33:03 may be a protective one in the Korean population. It was also found that A*02:07 is associated with skin lesions and arthritis, A*26:01 with uveitis, and A*30:04 with vascular lesions, genital ulcers, and positive pathergy test, independently of HLA-B*51. The meta-analysis performed in the present study confirmed that HLA-A*02:07 and A*26:01 are BD susceptibility alleles, whereas HLA-A*33:03 is associated with decreased risk of BD.

Although many studies investigated the HLA-class I region in BD patients, the majority reported insignificant results for *HLA-A *alleles; there was no significant *HLA-A *allele associated with BD in Palestine, Jordan, Iran, Ireland, Italy, and Turkey [27-31]. The low phenotypic frequencies of HLA-A*02:07, A*26:01, and A*30:04 in BD patients, which ranged between 5 and 15% in the present study, might have rendered it difficult to find associations between these *HLA-A *alleles and clinical manifestations in the previous studies that adopted a relatively small number of subjects. However, recent GWAS consistently showed that *HLA-A *region adds an independent contribution to the risk of BD [14-16].

The associations among HLA-A*02:07 and skin lesions and arthritis, and among HLA-A*30:04 and vascular lesions, genital ulcers, and positive pathergy test were revealed for the first time in the present study. Interestingly, not only HLA-A*02:07 but also HLA-B*51 appears to be a susceptibility allele for skin lesions (Table 4, Additional file 1). Furthermore, the majority of patients negative for both HLA-B*51 and HLA-A*02:07 exhibited skin lesions (Table 5), which suggests a large contribution of additional genetic loci to the skin manifestation of BD. Although HLA-A*30:04 was strongly associated with vascular lesions in the Korean population, no study subject carried the HLA-A*30:04 allele in the Japanese subjects [14,22,25,26] despite a high frequency of vascular involvement reported in Japanese BD patients [32]. These findings reveal a striking genetic difference, and we suggest that our result be compared with those obtained in other ethnic groups with sufficient HLA-A*30:04 carriers, if any. On the other hand, we are cautious to claim conclusively the specific associations between HLA-A*02:07 and arthritis or between HLA-A*30:04 and genital ulcers and a positive pathergy test, because patients without these clinical manifestations showed higher phenotypic frequencies of HLA-A*02:07 or A*30:04 than controls (Table 4). Moreover, these associations were not significant when patients with and without a particular clinical manifestation were compared. Therefore, there is a possibility that the above associations are merely due to increased disease susceptibility related to HLA-A*02:*07 *and A*30:04.

Elevated frequencies of HLA-A*26 have been reported in BD patients in Greece [19] and in patients with ocular manifestation in Taiwan [18]. HLA-A*26:01 not only has been reported to be a primary susceptibility allele of BD in Japan [14], but a recent study also found that the frequency of HLA-A*26:01 was significantly increased in BD patients with uveitis, particularly in the HLA-B*51 negative subset, in this ethnic group [33]. These findings are consistent with the present study. In addition, the decreased frequency of HLA-A*33:03 in BD patients in our study is consistent with the result obtained in the Japanese GWAS [14].

Although our results remain to be replicated in other cohorts, this is one of the few studies that comprehensively investigated the impact of the *HLA-A *gene on BD in relation to HLA-B*51. To avoid false negative results when assessing the association between certain *HLA-A *alleles and clinical manifestations of BD, we compared each clinical subset with a large number of controls. Then, patients with and without specific clinical manifestations were compared to validate the identified associations. Our results clearly show that certain *HLA-A *alleles are responsible for the unique clinical features of BD. The lack of individual demographic data of the controls might be one of the limitations of this study. Nevertheless, we believe that the results of our study are unlikely to be affected by systematic errors such as population stratification because the source of our controls, the unrelated hematopoietic stem cell donor registry of the KONOS, represents the whole Korean population rather than certain social groups within the population.

## Conclusions

This study investigated *HLA-A *alleles in BD patients and analyzed genetic susceptibilities to clinical manifestations of BD and found that HLA-A*02:07, A*26:01, and A*30:04 may be BD susceptibility alleles in the Korean population and are associated with skin lesions and arthritis, with ocular lesions, and with vascular lesions, genital ulcers, and positive pathergy test, respectively.

## Abbreviations

BD: Behcet's disease; CI: confidence intervals; GWAS: genome wide association studies; KONOS: Korean Network for Organ Sharing; OR: odds ratio; *Pc*: corrected *P*; PCR: polymerase chain reaction;

## Competing interests

The authors declare that they have no competing interests.

## Authors' contributions

EHK collected the clinical data, performed statistical analysis, and drafted the manuscript. JYK genotyped the *HLA *gene. FT helped design the study. JWK helped collect the clinical data. KS, EYL, YJL, EBL and MHP helped interpret the data. YWS was involved in the conception and design of the study. All authors read and approved the final manuscript.

## Supplementary Material

Additional file 1 The effect of HLA-B*51 on clinical manifestations of BD. Click here for file
