# Supplementary material for: Associations between the HLA-A polymorphism and the clinical manifestations of Behcet's disease
Source: Arthritis Res Ther. 2011 Mar 24;13(2):R49. doi: 10.1186/ar3292 (PMC3132038; doi:10.1186/ar3292)
Supplement: Additional file 1 — The effect of HLA-B*51 on clinical manifestations of BD. [file ar3292-S1.DOCX]

**Additional file 1.** The effect of HLA-B*51 on clinical manifestations of BD

| Group | Phenotype frequency of *B*51*  N (%) | OR [95% CI] | *P* |
| --- | --- | --- | --- |
| Patients with genital ulcers (N = 159) | 53 (33.3) |  |  |
| vs. Patients without genital ulcers (N = 64) | 28 (43.8) | 0.64 [0.36-1.16] | 0.14 |
| vs. Controls (N = 1398) | 282 (20.2) | 1.98 [1.39-2.82] | 0.0001 |
|  |  |  |  |
| **Patients with skin lesions (N = 180)** | 71 (39.4) |  |  |
| vs. Patients without skin lesions (N = 43) | 10 (23.3) | **2.15 [1.00-4.63]** | **0.047** |
| vs. Controls (N = 1398) | 282 (20.2) | **2.58 [1.86-3.57]** | **< 0.000005** |
|  |  |  |  |
|  |  |  |  |
| **Patients with uveitis (N = 85)** | 37 (43.5) |  |  |
| vs. Patients without uveitis (N = 138) | 44 (31.9) | **1.65 [0.94-2.88]** | **0.079** |
| vs. Controls (N = 1398) | 282 (20.2) | **3.05 [1.95-4.78]** | **< 0.00005** |
|  |  |  |  |
| Patients with vascular lesions (N = 33) | 9 (27.3) |  |  |
| vs. Patients without vascular lesions (N = 190) | 72 (37.9) | 0.61 [0.27-1.40] | 0.24 |
| vs. Controls (N = 1398) | 282 (20.2) | 1.48 [0.68-3.23] | 0.32 |
|  |  |  |  |
| Patients with positive pathergy test (N = 94) | 37 (39.4) |  |  |
| vs. Patients with negative pathergy test (N = 88) | 30 (34.1) | 1.26 [0.69-2.30] | 0.53 |
| vs. Controls (N = 1398) | 282 (20.2) | 2.57 [1.66-3.96] | < 0.00005 |
|  |  |  |  |
| Patients with arthritis (N = 125) | 42 (33.6) |  |  |
| vs. Patients without arthritis (N = 98) | 39 (39.8) | 0.77 [0.44-1.33] | 0.34 |
| vs. Controls (N = 1398) | 282 (20.2) | 2.00 [1.35-2.97] | < 0.0005 |
|  |  |  |  |

CI = confidence intervals; OR = odds ratio
